# 49 Temporal trends in ceftriaxone and cefepime susceptibility among Enterobacterales and parallel carbapenem use at a tertiary care center

**DOI:** 10.1017/ash.2026.10485

**Published:** 2026-06-23

**Authors:** Aarikha D’Souza, Joan Ivaska, Eric Luehr

**Affiliations:** 1 Banner Health

## Abstract

**Background:** Surgical Site Infections (SSI) are one of the most significant morbidities following surgical procedures, contributing substantially to prolonged length of stay, mortality and increased healthcare costs. Prevention bundles tailored to colon surgery have proven to be effective quality improvement interventions when implemented with high adherence, yet sustained compliance remains challenging across healthcare systems. **Methods:** A comprehensive colon-specific SSI prevention bundle was implemented across a large multi-state health system in 2019. The bundle encompasses perioperative interventions including preoperative measures (surgical antibiotic prophylaxis, mechanical and oral bowel preparations, appropriate hair removal, chlorhexidine bathing), intraoperative protocols (normothermia maintenance, chlorhexidine skin preparation, wound protectors, oxygen optimization), and closure techniques (separate sterile instrument sets, gown/glove changes, triclosan-coated sutures, silver dressings), with perioperative glycemic management maintained throughout. Bundle compliance was systematically monitored from 2019-2025, with SSI outcomes tracked using National Healthcare Safety Network (NHSN) criteria. Standardized Infection Ratios (SIRs) and compliance trends for key bundle elements were analyzed to assess intervention effectiveness. **Results:** From 2019-2025, implementation of the comprehensive SSI bundle across 14,355 colon procedures resulted in a 36.1% reduction in colon SIR (0.821 to 0.525). Significant improvements in key bundle compliance were observed: Surgical Antibiotic Prophylaxis (SAP) adherence increased 40.5% (41.9% to 82.4%), mechanical bowel preparation compliance rose 39% (64.2% to 89.3%), and oral bowel preparation adherence demonstrated a 119% increase (38.2% to 83.6%). Strong correlation was observed between bundle compliance rates and sustained SSI reduction, with the greatest improvements occurring in facilities achieving ?80% adherence with the SSI prevention bundle interventions. **Conclusion:** Sustained adherence to a comprehensive colon SSI prevention bundle significantly reduces postoperative infections, as demonstrated by a 36.1% SIR reduction over six years. Success requires systematic implementation, real-time compliance monitoring, institutional commitment to quality improvement, and ongoing evaluation of prevention bundle components to ensure adherence with current evidence-based national standards. The data suggest that optimizing SAP timing, mechanical bowel preparation, and oral antibiotic prophylaxis represent high-impact interventions warranting prioritization in resource-limited healthcare settings.

